# Applying Multiple Data Collection Tools to Quantify Human Papillomavirus Vaccine Communication on Twitter

**DOI:** 10.2196/jmir.6670

**Published:** 2016-12-05

**Authors:** Philip M Massey, Amy Leader, Elad Yom-Tov, Alexandra Budenz, Kara Fisher, Ann C Klassen

**Affiliations:** ^1^ Department of Community Health and Prevention Dornsife School of Public Health Drexel University Philadelphia, PA United States; ^2^ Division of Population Science Department of Medical Oncology Thomas Jefferson University Philadelphia, PA United States; ^3^ Microsoft Research Israel Herzeliya Israel; ^4^ Department of Biostatistics and Epidemiology Dornsife School of Public Health Drexel University Philadelphia, PA United States

**Keywords:** HPV vaccine, Twitter, communication methods, content analysis, data mining

## Abstract

**Background:**

Human papillomavirus (HPV) is the most common sexually transmitted infection in the United States. There are several vaccines that protect against strains of HPV most associated with cervical and other cancers. Thus, HPV vaccination has become an important component of adolescent preventive health care. As media evolves, more information about HPV vaccination is shifting to social media platforms such as Twitter. Health information consumed on social media may be especially influential for segments of society such as younger populations, as well as ethnic and racial minorities.

**Objective:**

The objectives of our study were to quantify HPV vaccine communication on Twitter, and to develop a novel methodology to improve the collection and analysis of Twitter data.

**Methods:**

We collected Twitter data using 10 keywords related to HPV vaccination from August 1, 2014 to July 31, 2015. Prospective data collection used the Twitter Search API and retrospective data collection used Twitter Firehose. Using a codebook to characterize tweet sentiment and content, we coded a subsample of tweets by hand to develop classification models to code the entire sample using machine learning procedures. We also documented the words in the 140-character tweet text most associated with each keyword. We used chi-square tests, analysis of variance, and nonparametric equality of medians to test for significant differences in tweet characteristic by sentiment.

**Results:**

A total of 193,379 English-language tweets were collected, classified, and analyzed. Associated words varied with each keyword, with more positive and preventive words associated with “HPV vaccine” and more negative words associated with name-brand vaccines. Positive sentiment was the largest type of sentiment in the sample, with 75,393 positive tweets (38.99% of the sample), followed by negative sentiment with 48,940 tweets (25.31% of the sample). Positive and neutral tweets constituted the largest percentage of tweets mentioning prevention or protection (20,425/75,393, 27.09% and 6477/25,110, 25.79%, respectively), compared with only 11.5% of negative tweets (5647/48,940; *P*<.001). Nearly one-half (22,726/48,940, 46.44%) of negative tweets mentioned side effects, compared with only 17.14% (12,921/75,393) of positive tweets and 15.08% of neutral tweets (3787/25,110; *P*<.001).

**Conclusions:**

Examining social media to detect health trends, as well as to communicate important health information, is a growing area of research in public health. Understanding the content and implications of conversations that form around HPV vaccination on social media can aid health organizations and health-focused Twitter users in creating a meaningful exchange of ideas and in having a significant impact on vaccine uptake. This area of research is inherently interdisciplinary, and this study supports this movement by applying public health, health communication, and data science approaches to extend methodologies across fields.

## Introduction

Human papillomavirus (HPV) is the most common sexually transmitted infection in the United States [1]. In the United States, approximately 79 million people are infected with HPV, and 14 million will become newly infected each year [2,3]. Although many infections will resolve without serious consequences, HPV infection has been causally linked to cervical and anal cancers, as well as genital warts. Several HPV vaccines are licensed in the United States that protect against strains of HPV most associated with cervical cancer in females and genital warts in males [4]. Thus, HPV vaccination has become an important component of adolescent preventive health care. According to the US Centers for Disease Control and Prevention, in the United States, HPV vaccination rates have steadily increased among adolescent girls yet remain lower in adolescent boys. In 2007, among girls aged 13-17 years, only 25.1% initiated the vaccine series and 5.9% completed the series, compared with 60.0% initiation and 39.7% completion in 2014 [5]. In 2014, vaccination rates among boys aged 13-17 years were 41.7% initiation and 21.6% completion [5].

A substantial body of communication research demonstrates that mediated communication reflects, but also serves to shape, popular understanding of important issues, including health [6-10]. As media evolves, more information about HPV vaccination is shifting to digital platforms on the Internet, in the form of websites, personal blogs, and social media. Of particular concern is the accuracy or viewpoint of the information. An analysis of the top search results about the HPV vaccine returned from Google, Yahoo, Bing, and Ask.com found that, while the majority of websites (57%) maintained a neutral tone about vaccination, a significant number of sites contained inaccuracies or mentioned conspiracy theories about the vaccine [11]. Users on Twitter, a popular microblogging social media platform, communicate about a range of topics, and there is strong evidence that communication includes a sizeable discourse on public health research and practice, including surveillance [12-16] and information dissemination [17-19].

Health information consumed on social media may be especially influential for segments of society such as younger populations, as well as ethnic and racial minorities, who may be less likely to access health information through formal news sources, health care providers, and other more traditional resources. For example, Latino, African American, and younger populations are more likely than white and older respondents to use mobile technologies for health information [20]. Furthermore, young users, along with minority users, disproportionately access Twitter on mobile devices. This is no surprise, as minority audiences are among the highest users of mobile technologies and social networking platforms [21,22].

A major motivation for this study was the evolving nature of Web-based health information and the opportunity to better understand this area of inquiry through interdisciplinary research. That is, the ways in which Web-based health information is searched for and consumed are no longer limited to a 1-way or static process (ie, using a search engine). Rather, more and more Web information seekers are turning to more dynamic informational sources, including social media, blogs, and forums, to access but also respond to health information [23]. A recent nationwide survey conducted by the Pew Research Center, focusing on how adults in the United States use Web-based resources for health information, found that among Web-based health information seekers, 16% tried to find others who might share the same health concerns, 30% consulted Web-based reviews or rankings of health care services or treatments, and 26% read or watched someone else’s experience about a health issue [23]. Instinctively, public health researchers and practitioners are beginning to examine how health information is generated and disseminated via Twitter. There is a growing evidence base detailing methods for data collection and analysis using social media platforms, and in this study we sought to further this literature by describing a novel approach to data collection using two data collection tools.

These types of analyses and studies are particularly useful for the public health community, in tracking the dissemination of information about vaccination across populations and to gauge receptivity to vaccination messages. Researchers are just beginning to assess the extent and type of discourse about the HPV vaccine on Twitter, although methods are varied [24-27]. This emerging area of communication research provides an opportunity for interdisciplinary teams among the fields of public health, health communication, and data science to strengthen the science and methodology in this growing area of research. Thus, the purposes of this study were to quantify HPV vaccine communication on Twitter, specifically focusing on (1) sentiment, (2) side effects, and (3) prevention and protection, and to describe a novel methodology using two data collection methods to analyze Twitter data.

## Methods

**Figure 1:**
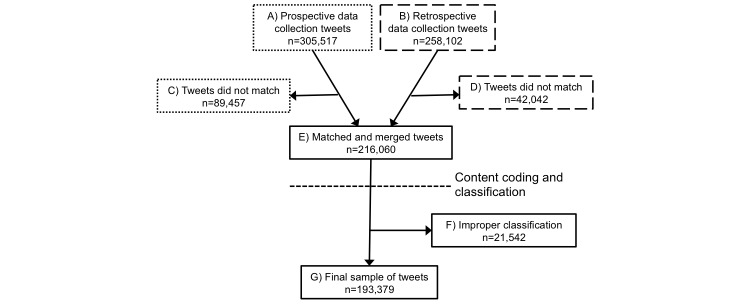
Flowchart detailing data collection, merging, and cleaning of final dataset of tweets related to human papillomavirus vaccination.

We used two methods to collect and validate Twitter data related to HPV vaccination, detailed in Figure 1. The first method used prospective data collection (Figure 1, box A) with a proprietary software program developed by Black and colleagues [28], and the second method employed retrospective data collection (Figure 1, box B) through Microsoft Research. To identify tweets related to HPV vaccination, we used the following keyword search terms and hashtags: “HPV,” “#HPV,” “HPV vaccine,” “#HPVvaccine,” “HPV shot,” “#hpvshot,” “Gardasil,” “#Gardasil,” “Cervarix,” and “#Cervarix.” We developed these keywords by drawing from previous research in content analyses of HPV print and Web-based news sources, balancing the general HPV-related topics with vaccine-specific information [29,30].

### Prospective Data Collection

The proprietary software was developed to capture Twitter messages in real time, or prospectively, based on keyword search terms. Using the Twitter Search API (Twitter, Inc), the software collected publicly available data that contained tweets that were 6 or fewer days old, although it may include tweets up to 9 days old. The software captured not only the message posted on Twitter, including hashtags (#) and mentions (@), but also features of the message, such as information about the Twitter user; the date, location, and language of the tweet; and the number of times the message was retweeted. Information on the software architecture is detailed elsewhere [28]. As the software used the Twitter Search API, we used the software to conduct prospective data collection at least once a week during the study period (August 1, 2014 to July 31, 2015). We collected a total of 305,517 tweets using prospective data collection.

### Retrospective Data Collection

In August 2015, after completing prospective data collection, Microsoft Research accessed Twitter Firehose to extract data using the same keyword search terms. Twitter Firehose allowed access to all tweets during the study timeline (August 1, 2014 to July 31, 2015), except those deleted by the users. We collected a total of 258,102 tweets using retrospective data collection.

### Data Integration and Validation

All tweets contained a unique tweet identification (ID) number. Using this unique tweet ID, we merged and matched the prospective and retrospective datasets to produce a final dataset of n=216,060 tweets (Figure 1, box E). We included retweets in this final dataset as long as they had a unique tweet ID. The final dataset included 71.72% (89,457/305,517) of the prospective data and 83.71% of the retrospective data (42,042/258,102). Tweets that did not match were excluded from the final dataset (Figure 1, boxes C and D). Possible reasons for tweets not matching from the two data sources were that (1) user-selected language preferences changed between the prospective and retrospective collection periods, and thus the language variable did not match, (2) gaps longer than 7 days in prospective data collection due to study team scheduling would result in missing tweets no longer available through the Twitter Search API, and (3) some tweets had been deleted. Importantly, deleted tweets or tweets from suspended users that were captured in the prospective data collection were omitted from the final sample because they would not have been captured in the retrospective data collection (per Twitter’s user and data policy). We included English-language tweets, regardless of specific location, in the study; we removed tweets in all other languages from the final dataset.

### Content Coding

Data captured included both the tweet content or message itself, contained within 140 characters, and information on the characteristics of the communication and sender. We developed a codebook to classify the content of the 140 characters, and in this study we report on sentiment toward the HPV vaccine (positive, negative, neutral, or no mention), side effects discussed, and prevention or protection discussed. We derived the coding system from previous content analysis research conducted by study team members about the HPV vaccine [29,30], although in print rather than in social media, as well as published Twitter content analysis research [13]. Table 1 details the sample codebook with features including variable description, along with tweet examples. “No mention” is included as part of sentiment because we wanted to situate vaccine sentiment within the larger HPV communication environment on Twitter. Therefore, we are able to quantify how much of the HPV discussion was vaccine focused and how this compares with HPV communication in general.

**Table 1:** Content classification codebook with feature description and tweet example for tweets related to human papillomavirus (HPV) vaccination.

Feature		Description	Example tweet
**Sentiment**				
	Positive	The tweet contains supportive messages about the HPV vaccine and encourages its uptake	1. Not only does the HPV vaccine protect against human papillomavirus, but it also reduces the risk of cancers 2. #HPV vaccine can be #cancer prevention! Parents, #vaccinate your children at ages 11-12
	Negative	The tweet contains disparaging messages about the HPV vaccine or discourages its uptake	1. Healthy 12-year-old girl dies shortly after receiving HPV vaccine 2. RT^a^ @CBCHealth: The Gardasil Girls: How Toronto Star story on young women hurt public trust in vaccine http://t.co/...
	Neutral	The tweet’s text holds no subjective opinions about the vaccine—purely facts repeated from sources	1. State officials unveil campaign for HPV vaccination http://t.co/0I2sAWGXYs 2. RT @DrJenGunter: About 10% boys have received 3 doses HPV vax
	No mention	The tweet does not mention the HPV vaccine	1. RT @Forbes: HPV is truly indiscriminate 2. RT @CDCSTD: #Women: get screened & talk w/ your friends about the link between #HPV & cervical #cancer
Side effects		The tweet refers to side effects caused by the HPV vaccine or effects that may be unknown to the user	1. Healthy 12-year-old girl dies shortly after receiving HPV vaccine 2. RT @ksbrowneyedgirl: It can happen to your child...to your family...#OneLess #Gardasil #CDCwhistleblower #vaccine...
Prevention/protection		The tweet refers to the extent to which the HPV vaccine will protect the user from or prevent negative health outcomes	1. Single HPV jab could prevent 70% of cervical cancers (http://t.co/Hg0KSlIk2A) 2. A new HPV vaccine prevents nine strains of the virus http://t.co/ZFGvVqlq0U

^a^RT: retweet.

Next, 5 members of the team independently coded the same random sample of tweets (n=50) for each of the codebook variables. Interrater reliability was high (>0.8 on each variable) among all 5 coders, indicating that the codebook is systematic and replicable. Based on developed coding procedures, 2 study members manually coded additional tweets for the purpose of developing classification models. This was an iterative process that involved manual coding of tweets, then computer-assisted coding, followed again by manual coding to refine the classification results. After each round of coding, we analyzed randomly selected tweets to validate model classifications. In total we coded 1470 tweets manually over 4 iterative rounds.

We used the 1470 manually coded tweets to develop a machine learning classifier for each variable in the codebook. Binary variables were classified using a linear classifier (Moore-Penrose pseudoinverse), while a decision tree was applied to variables with more than two categorical responses. Multimedia Appendix 1 details features of the classifiers.

**Figure 2:**
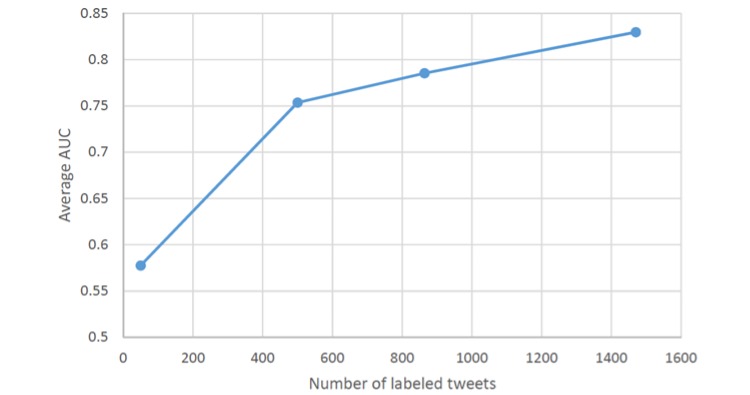
Average area under the receiver operating characteristic curve (AUC) as a function of manually coded tweets.

We evaluated the classifiers using leave-one-out estimation; that is, we trained classifiers on (*n* –1) samples and tested them on the remaining sample, repeating the process *n* times without replacement [31]. The accuracy of the classifications for binary variables was evaluated by the area under the receiver operating characteristic curve (AUC) and multiple-valued attributes via the fraction of errors. Figure 2 shows that the average AUC improved as a function of the number of manually coded tweets. In this study, we investigated 3 variables classified through this process: HPV vaccine sentiment (AUC=0.918), side effects (AUC=0.739), and prevention/protection (AUC=0.774).

After evaluating the performance of the machine classifiers, we then applied these to the full set of 216,060 tweets in the study, except for the 1470 manually labeled tweets. Based on the coding scheme, no study variables should have classification values rounding to zero; however, this was the case for some of values, indicating improper classification based on the coding scheme. Therefore, we randomly sampled 300 tweets with rounded classification values of zero and discerned no clear pattern within any of the unrounded classification values. That is, the tweets with values rounding to zero were not consistently supposed to be coded as 1 or another value. Due to this inconsistent classification pattern and to provide conservative estimates, we discarded all observations with a rounded classification of zero for sentiment, side effects, and prevention/protection (Figure 1, box F), leaving us with a final study sample of n=193,379 (Figure 1, box G).

Finally, for each tweet collected, we documented the words in the 140-character tweet text most associated with each keyword. We did this by computing the probability that a word would appear in tweets that contain the keyword, compared with the probability of that word in the entire corpus of tweets (n=193,379). To compute the probability, we counted the number of times each word appeared in the given set of tweets and divided this count by the total number of words in the set.

We used the statistical package Stata 14 (StataCorp LP) to analyze differences in tweet characteristics by tweet sentiment. To determine significant differences by sentiment, we used chi-square tests for the counts and categorical variables, analysis of variance for the continuous variables, and nonparametric equality of medians for the medians.

## Results

**Figure 3:**
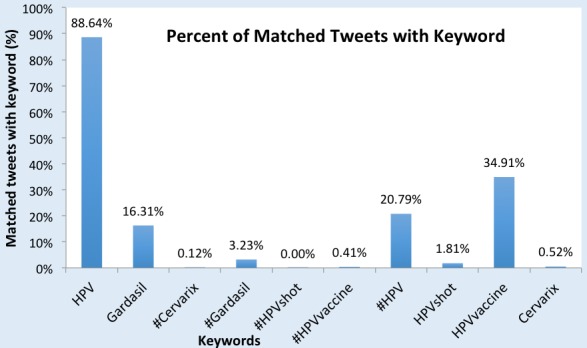
Percentage of tweets in the final sample by keyword search.

A total of n=193,379 English-language tweets were collected, classified, and analyzed between August 1, 2014 and July 31, 2015. Figure 3 details the percentage of tweets that included the specified keyword search term. The keyword categories are not mutually exclusive, and the same tweet could be captured in multiple keyword searches. In our final dataset, the potential overlap of tweets across keyword searches was reconciled during the deduping and merging process. Over 88.64% (191,515/216,060) of the final dataset included the keyword search term HPV, and nearly 34.91% (75,433/216,060) included HPV vaccine. Based on the keyword query process, all tweets captured by the keyword HPV vaccine would by definition also be captured by the keyword HPV, as both searches share HPV.

Table 2 displays the words most associated with each keyword search term. Results show that associated words varied with each keyword, with HPV being associated with personal words such as *I, me,* and *have*, and #HPV being associated with *January* (cervical cancer awareness month), *prevent*, and *learn*. Words associated with the specific vaccine-related keywords (ie, HPV vaccine, Gardasil, and Cervarix) varied greatly, with more positive and preventive words being associated with HPV vaccine (ie, *read*, *to prevent*, *for girls*), and more negative words being associated with Gardasil and Cervarix (ie, *cdcwhistleblower*, *exposed*).

**Table 2:** Most significant terms associated with each keyword in tweets related to human papillomavirus (HPV) vaccine.

Keyword	Most significant terms
HPV	me, my, have HPV, I, I have, been, got, read, have to, like
#HPV	been, cervicalcancer, disease, January, to prevent, just, learn, it’s, vaccine to, time, out
HPV vaccine	read, been, to prevent, vaccine to, I, out, for girls, age, the HPV, HPV vaccine
#HPVvaccine	HPVvaccine, to have, please, cancer is, teens, vaccine for, cervicalcancer, linked to, getting, safe
HPV shot	shot, have to, got, my, I have, me, go, to get, like, I
#HPVshot	12 year, 13 year, a new, about, about HPV, about the, active, after, against, against HPV
Gardasil	shot, Gardasil, 13 year, me, cdcwhistleblower, my, want, I, have to, got
#Gardasil	13 year, cdcwhistleblower, Gardasil, HPVvaccine, 100, need to, her, think, life, please
Cervarix	exposed, need to, many, medical, need, to be, cdcwhistleblower, health, how, research
#Cervarix	exposed, need to, medical, many, need, to be, cdcwhistleblower, health, research, link between

Table 3 describes overall sample totals, as well as sample totals by vaccine sentiment in the tweet (positive, negative, neutral, and no mention). Between August 2014 and July 2015 there were a total of 78,643 unique users who tweeted about HPV, with an average of 2.5 tweets per user. The average number of followers per user was 6569, compared with the median number of followers per user of 443. This large difference between the average and median number of followers indicates that, in our sample, the number of followers per user was heavily skewed to the right (toward a small number of highly followed users). Moreover, the number of followers differed significantly by sentiment (*P*<.001), with a greater average number of followers exposed to positive sentiment than to negative sentiment (8022 vs 4772, respectively). A majority of tweets included a URL (138,059/193.379, 71.39%), nearly half included a hashtag (86,966/193.379, 44.97%), and just over half included a mention (112,049/193.379, 57.94%). Additionally, there were an average of 12 retweets per tweet and a median of 1 retweet per tweet. Tweets that did not mention the HPV vaccine had the highest average retweet count of 22 retweets per tweet.

**Table 3:** Characteristics based on metadata and classification analysis, n=193,379.

Characteristic		Tweet sentiment toward vaccine				Total	*P* value	
		Positive	Negative	Neutral	No mention^a^			
**HPV vaccine tweets**							
	Total tweets, n	75,393	48,940	25,110	43,936	193,379	<.001
	Tweets with sentiment, %	38.99	25.31	12.98	22.72	—	
**Users**							
	Total users, n	36,283	24,010	15,045	25,954	78,643^b^	<.001
	Average HPV vaccine tweets per user	2.1	2.0	1.7	1.7	2.5	<.001
	Average followers per user	8022	4772	6093	6352	6569	<.001
	Median followers per user	459	467	445	381	443	<.001
**Tweet contents**							
	Includes at least 1 link, n	57,800	34,491	18,898	26,870	138,059	<.001
	Link, %	76.66	70.48	75.26	61.16	71.39	
	Includes at least 1 hashtag, n	36,638	21,523	10,890	17,915	86,966	<.001
	Hashtag, %	48.60	43.98	43.37	40.78	44.97	
	Includes at least 1 mention, n	44,558	31,085	12,659	23,747	112,049	<.001
	Mention, %	59.10	63.52	50.41	54.05	57.94	
	Mentions prevention/protection, n	20,425	5647	6477	3209	35,758	<.001
	Prevention/protection, %	27.09	11.54	25.79	7.30	18.49	
	Mentions side effects/unknowns, n	12,921	22,726	3787	2619	42,053	<.001
	Side effects/unknowns, %	17.14	46.44	15.08	5.96	21.75	
**Retweets**							
	Average number of retweets per tweet	9.7	9.1	7.8	22.0	12.1	<.001
	Median number of retweets per tweet	1	1	0	0	1	<.001

^a^No mention of HPV vaccine, but mention of HPV.

^b^Total differs from sum of totals because some users tweeted in multiple categories.

Table 3 also displays results by tweet sentiment. Positive, negative, or neutral sentiment describes how the HPV vaccine was communicated, and no mention indicates that the HPV vaccine *was not mentioned* (thought HPV *was mentioned*). Positive sentiment toward the vaccine was the largest type of sentiment in the sample, with 75,393 positive tweets (38.99% of the sample). Negative sentiment was the second largest type with 48,940 tweets (25.31% of the sample). Many more users participated in positive sentiment than in negative sentiment (36,283 vs 24,010 users, respectively).

Tweets coded as having positive sentiment toward HPV vaccine and no mention of HPV vaccine had a significantly higher use of URLs (57,800/75,393, 76.66%, and 18,898/25,110, 75.26% containing links, respectively) as compared with negative sentiment (34,491/48,940, 70.48%) (*P*<.001). The use of hashtags and mentions in tweets was fairly consistent across sentiment, with positive sentiment showing the greatest use of hashtags (36,638/75,393, 48.60%) and negative sentiment showing the greatest use of mentions (31,085/48,940, 63.52%).

When examining sentiment by tweet content, positive and neutral tweets had the largest percentage of mentions of prevention/protection (20,425/75,393, 27.09%, and 6477/25,110, 25.79%, respectively), compared with only 11.54% (5647/48,940) of negative tweets (*P*<.001). Nearly one-half (22,726/48,940, 46.44%) of negative tweets mentioned side effects, compared with only 17.14% (12,921/75,393) of positive tweets and 15.08% (3787/25,110) of neutral tweets.

## Discussion

This study took place at the intersection of public health, health communication, and data science, and demonstrated the application of a novel methodology for collecting and integrating Twitter data from multiple sources, as well as supporting prior research showing the use of content and classification analysis. Building upon prior studies, we demonstrated the application of similar analyses and techniques across health domains [25,32], as well as expanding data collection and integration procedures. Using tweet characteristics, as well as content analysis based on classification models, we examined the potential reach and classified the nature of HPV vaccine-related tweets from August 2014 to July 2015 (n=193,379).

As our results show, words matter from both a data collection standpoint and a content perspective. The vast majority of our data contained the keyword HPV (88.64%), and thus future studies may be able to limit their keyword search to this single word, especially if resources are limited. Additionally, our findings show that different keywords are associated with different word clusters. HPV, for example, was associated with personal words such as *me, I,* and *have*, whereas #HPV was associated with more awareness-raising words, such as *January*, *prevent*, and *learn*. The hashtag (#) is an important feature on Twitter that categorizes tweets based on keyword and makes it easier to search other tweets with that same hashtag and keyword. By including #HPV in a tweet, users are able to click on #HPV and read other messages that have also included #HPV, thus acting as a “social search” function. This search feature may play an important role in raising awareness, as demonstrated by the associated word clusters. Importantly, brand-specific HPV vaccines were associated with more negative words on Twitter (ie, *cdcwhistleblower*, *exposed*), whereas the general keyword HPV vaccine was not. This could be important to understand when crafting messages about HPV and the vaccine: including brand-specific vaccines may encourage or lead to a more negative space in Twitter conversations.

Despite some research demonstrating that Web-based vaccine information can have a largely negative sentiment [11], our findings show that a great percentage of tweets about HPV vaccine had a positive sentiment, helping to validate findings on the same topic [24-27]. Furthermore, over one-quarter of these positive HPV vaccine tweets mentioned prevention or protection. In addition, positive tweets had, on average, many more followers than negative tweets, indicating the potential for a greater reach and more exposure of positive tweets than of negative tweets.

There is also an important relationship between tweet sentiment and tweet content: many more tweets that were classified as positive mentioned information about prevention or protection, whereas tweets classified as negative included a much greater discussion about side effects. This can be important information for health promotion and communication campaigns, specifically in terms of tailoring a message and joining a particular conversation. As tweets that contain information on side effects are more likely to be part of a negative conversation, tweeting “side effects are minimal,” that is, downplaying negative sentiment, may not be the most effective way to communicate this information. Importantly, for the “no mention” category (only discussion of HPV and not the vaccine), there was a very low percentage of tweets that mentioned prevention or protection as well as side effects. This is an important data analysis check in terms of the validity of our classification models, as we would not expect a large percentage of this content because it relates almost exclusively to the vaccine.

On Twitter, the distribution of followers by user is often highly skewed to the right; that is, few users (often dubbed the elite users, such as celebrities, organizations, and other high-profile users) have many more followers than the majority of Twitter users [33,34]. Our data support this distribution, as the average number of followers per user is much higher than the median number of follower per user (6569 compared to 443). When comparing average number of followers by sentiment, our findings show that tweets with positive sentiment toward the vaccine had a much higher average follower count than did tweets with negative sentiment, averaging 8022 followers compared to only 4772 followers, respectively. This suggests that more influential users, as measured by the number of followers, are tweeting about the vaccine more positively than negatively. This is an important finding, as number of followers is a proxy for reach, and thus more Twitter users are potentially being exposed to more positive sentiment than negative [33,34]. However, when examining sentiment distribution by the median, the numbers of followers are much closer, at 459 (positive) and 467 (negative). This suggests a more evenly distributed reach, or exposure, of positive and negative sentiment among typical Twitter users.

Social features on Twitter, including hashtags, mentions, and links, are important features to help with message dissemination and reach. Tweets classified as having positive sentiment accounted for the greatest percentage of tweets with links: 76.66% of positive tweets contained at least one link. Links are used to connect to other Web sources, often increasing the likelihood of interactivity (ie, shared by a retweet) and serving as a source information to support and corroborate the veracity of tweet content [35]. Tweet link content needs to be examined in order to examine the nature of links. Conversely, tweets classified as having negative sentiment had the greatest percentage of mentions per tweet: 63.52% (31,085/48,940) of negative tweets contained at least one mention. Mentions are a way to communicate directly to other users (more directed messaging and communication), and may serve as a way for agenda setters and opinion leaders to emerge in a network [36], bringing more attention to themselves and their message [37], and to control message diffusion [38]. Use of mentions may be a mechanism for negative and alternative messages about the HPV vaccine, which are in the minority, to appear to gain clout and recognition.

Measuring retweets is a way to quantify reach and dissemination [34,37,38], and in our sample “no mention” had the highest average number of retweets per tweet. “No mention” captures messages about HPV in general and not specifically related to HPV vaccine. This could potentially indicate an interest in disseminating information about the virus in general, or could be related to cervical cancer awareness and screening.

Understanding the content and implications of conversations that form around HPV vaccination on social media can aid health organizations and health-focused Twitter users in creating a meaningful exchange of ideas and having a significant impact on vaccine uptake. As HPV vaccination campaigns continue to use social media platforms, it is important to understand trends in social media communication, particularly across media platforms. In terms of public health surveillance, our study demonstrated that, despite an often negative-leaning frame and discussion of HPV vaccine on social media, the greatest percentage of HPV vaccine tweets are positive. Understanding effective dissemination channels will help connect campaigns with “elite” users and media who have many followers, and consequently may lead to a wider reach of message.

### Limitations

While this study contributes to interdisciplinary research and methods, there are a few limitations worth noting. First, when merging tweets from the two data sources, we excluded 29.28% (89,457/305,517) of the prospective data and 16.29% (42,042/258,102) of the retrospective data due to nonmatching tweet IDs. To investigate why tweet IDs did not match and were thus excluded from the final sample, we examined excluded tweets from both data collection methods. We randomly selected 1000 tweets from the prospective data, which we identified through Twitter Search API, and found that many were excluded due to English-language misclassification (456/1000, 45.6%), deletion of the tweet (324/1000, 32.4%), no keyword being matched in the text (78/1000, 7.8%), and unexplained reasons (142/1000, 14.2%). We examined all tweets from the retrospective data, which were identified through Twitter Firehose, and found that many were excluded due to gaps in time of the prospective data (9821/42,042, 23.36%) and a majority for unexplained reasons (32,221/42,042, 76.64%).

While excluding tweets from the final sample may be a limitation, our study shows that the majority of tweets captured using the Twitter Search API, which is accessible to the public, was validated against the gold standard of Twitter Firehose. According to Twitter, Firehose contains “all public statuses,” compared with Twitter Search API, which only “offer samples of the public data flowing through Twitter.” Twitter Search API is further limited by the number of queries that can be made to it and the number of responses it returns. Therefore, for example, if there is a surge in the use of a keyword, only some of the tweets using it will be returned using Twitter Search API. For this reason, Firehose is considered the gold standard for Twitter data collection.

Second, when applying the classification models to our sample, we may have misclassified some of the tweets or not classified some at all. To limit misclassification, we used an iterative process that included multiple rounds of human coding to strengthen the computer classification models, and we reached on average 80% accuracy with each model. Tweets that were not classified with an adequate level of certainty (ie, above 70%) were excluded from the final sample. This process allowed for our entire sample of tweets to be classified as opposed to a randomly selected subsample.

Third, when discussing reach and exposure of health messages on Twitter, it is important to note that, when a user tweets, it does not mean that all of their followers will read the tweet. Thus, it is most accurate to refer to potential reach and exposure, as opposed to actual reach. In addition, analyzing the follower network of each user and tweet would provide additional information on reach; however, that was beyond the scope of this study.

### Conclusion

Using and leveraging social media to detect health trends, as well as communicate important health information, is a growing area of research in public health. This area of research is inherently interdisciplinary, and this study supports this movement by applying public health, health communication, and data science approaches to extend methodologies across fields. Building on this particular study, future research will need to further examine how various stakeholders, including parents, youth, health care providers, and health care systems, communicate about the HPV vaccine and identify opportunities to strengthen vaccine uptake and completion. Furthermore, identifying how communication trends are associated with behavioral outcomes, that is, actual vaccine uptake, will be an important next phase of this area of inquiry.

## Appendix

Multimedia Appendix 1 Features of the classifiers.

## Abbreviations

AUC: area under the receiver operating characteristic curve
HPV: human papillomavirus
ID: identification
