# Supplementary material for: Applying Multiple Data Collection Tools to Quantify Human Papillomavirus Vaccine Communication on Twitter
Source: J Med Internet Res. 2016 Dec 5;18(12):e318. doi: 10.2196/jmir.6670 (PMC5168526; doi:10.2196/jmir.6670)
Supplement: Multimedia Appendix 1 [file jmir_v18i12e318_app1.pdf]

Multimedia Appendix 1
Features of the classifiers

Tweet text was converted to lower case before processing. We removed URLs from tweet text and handled them separately (attributes 7 and 8).

Features for the classifiers:

1. Word and word pairs that appeared in at least 10 words.
2. Whether the tweet was a copy of another tweet (retweet)
3. Whether the tweet mentioned a specific user
4. Whether the tweet included a question mark
5. Whether the tweet included a hash tag
6. The number of words in the tweet
7. Whether the tweet included a URL
8. The top and second level URL (of an expanded URL), if present
9. User attributes:
    a. Words and word pairs from the user’s description (User Bio)
    b. Whether the user description included a URL
    c. Whether the user was verified
    d. Whether the user gave his location
